# Drier tropical and subtropical Southern Hemisphere in the mid-Pliocene Warm Period

**DOI:** 10.1038/s41598-020-68884-5

**Published:** 2020-08-10

**Authors:** Gabriel M. Pontes, Ilana Wainer, Andréa S. Taschetto, Alex Sen Gupta, Ayako Abe-Ouchi, Esther C. Brady, Wing-Le Chan, Deepak Chandan, Camille Contoux, Ran Feng, Stephen J. Hunter, Yoichi Kame, Gerrit Lohmann, Bette L. Otto-Bliesner, W. Richard Peltier, Christian Stepanek, Julia Tindall, Ning Tan, Qiong Zhang, Zhongshi Zhang

**Affiliations:** 1 Oceanographic Institute, University of São Paulo, São Paulo, Brazil; 2 Climate Change Research Centre, The University of New South Wales, Sydney, Australia; 3 ARC Centre of Excellence for Climate Extremes, The University of New South Wales, Sydney, Australia; 4 Centre for Earth System Dynamics, Atmosphere and Ocean Research Institute, University of Tokyo, Tokyo, Japan; 5 National Center for Atmospheric Research, Boulder, USA; 6 Department of Physics, University of Toronto, Toronto, Canada; 7 Laboratoire des Sciences du Climat et de l’Environnement, Université Paris-Saclay, 91191 Gif-sur-Yvette, France; 8 School of Earth and Environment, University of Leeds, Leeds, UK; 9 Faculty of Life and Environmental Sciences, University of Tsukuba, Tsukuba, Japan; 10 Alfred Wegener Institute, Helmholtz Centre for Polar and Marine Research, Bremerhaven, Germany; 11 Key Laboratory of Cenozoic Geology and Environment, Institute of Geology and Geophysics, Chinese Academy of Sciences, Beijing, China; 12 Department of Physical Geography and Bolin Centre for Climate Research, Stockholm University, Stockholm, Sweden; 13 NORCE Norwegian Research Centre, Bjerknes Centre for Climate Research, Bergen, Norway

**Keywords:** Climate sciences, Atmospheric science, Climate change, Palaeoclimate

## Abstract

Thermodynamic arguments imply that global mean rainfall increases in a warmer atmosphere; however, dynamical effects may result in more significant diversity of regional precipitation change. Here we investigate rainfall changes in the mid-Pliocene Warm Period (~ 3 Ma), a time when temperatures were 2–3ºC warmer than the pre-industrial era, using output from the Pliocene Model Intercomparison Projects phases 1 and 2 and sensitivity climate model experiments. In the Mid-Pliocene simulations, the higher rates of warming in the northern hemisphere create an interhemispheric temperature gradient that enhances the southward cross-equatorial energy flux by up to 48%. This intensified energy flux reorganizes the atmospheric circulation leading to a northward shift of the Inter-Tropical Convergence Zone and a weakened and poleward displaced Southern Hemisphere Subtropical Convergences Zones. These changes result in drier-than-normal Southern Hemisphere tropics and subtropics. The evaluation of the mid-Pliocene adds a constraint to possible future warmer scenarios associated with differing rates of warming between hemispheres.

## Introduction

**Figure 1:**
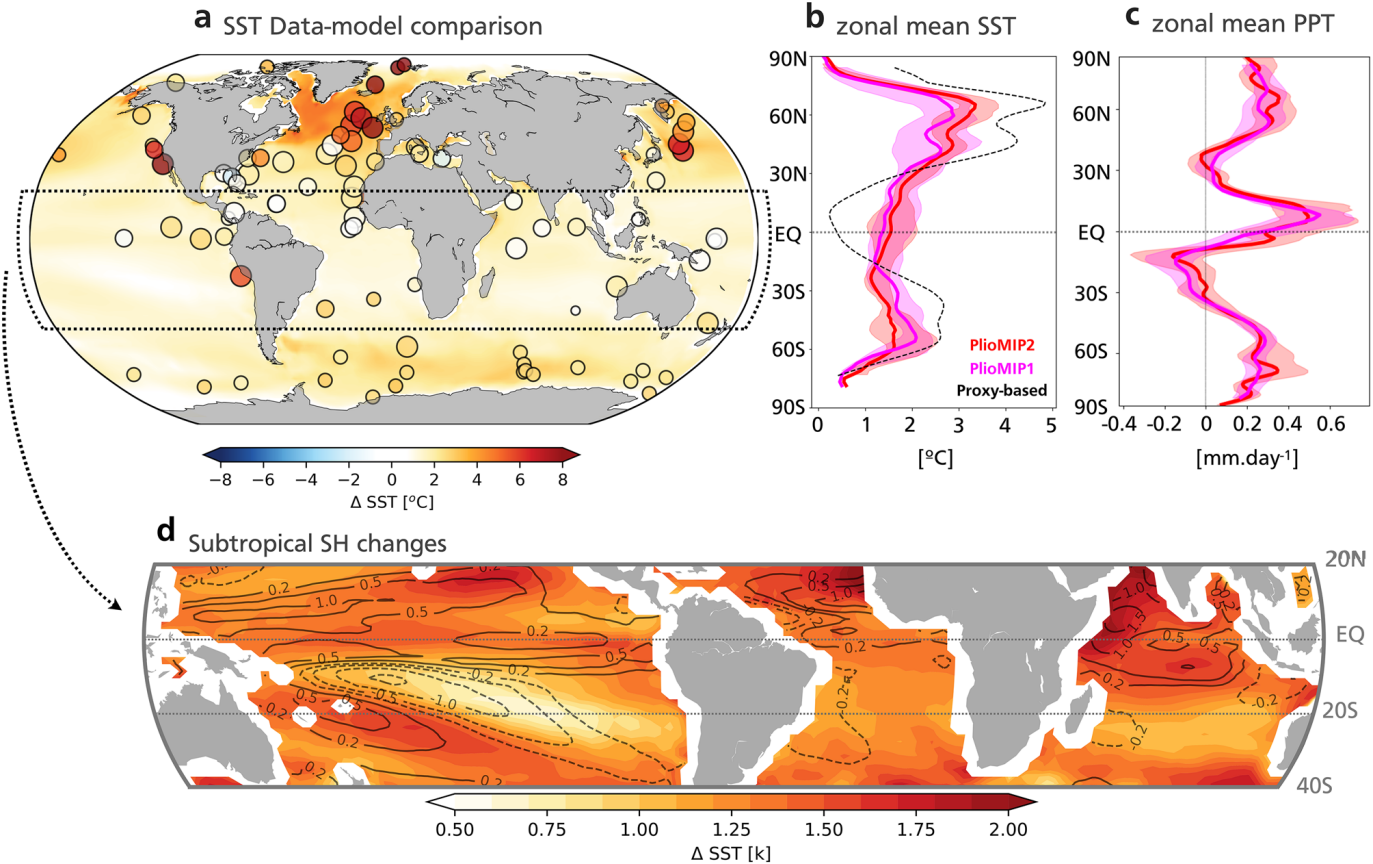
Data-model comparison of Sea Surface Temperatures anomalies during the mid-Pliocene and PlioMIP precipitation. (**a)** Multi-model mean SST anomaly in the PlioMIP2 (mid-Pliocene minus pre-industrial). Circles indicate location of sites and SST anomalies compiled by PRISM^11^. Size of the circles indicate confidence level. Small: low confidence. Medium: medium confidence. Large: High confidence. (**b)** zonal mean SST anomaly from PRISM (dashed black) and multi-model medians PlioMIP1 (magenta) and PlioMIP2 (red). (**c)** as per ‘b’ but for precipitation. Banding indicates interquartile range. d) as per ‘a’ but including contours (black) of precipitation changes in mm.day^−1^.

The Earth has experienced many periods in which climate was warmer than present. Understanding atmospheric circulation and precipitation during past warm climates is useful to produce constraints on possible future changes. Here we analyse the Southern Hemisphere large-scale rainfall response in the mid-Pliocene Warm Period (~ 3 Ma mPWP; hereafter referred as mid-Pliocene). During this period high-latitude Sea Surface Temperatures (SST) were as high as + 9 °C and + 4 °C in the Northern and Southern Hemisphere, respectively^1^, compared to pre-industrial times (~ 1850 Common Era [C.E.]; Fig. 1). In addition, the Greenland ice sheet had 50–70%^2–4^ less mass and the western Antarctica was ice-free^5,6^. Atmospheric CO_2_ concentrations were similar to today^7^ (~ 400 ppm). Although the extent of the ice sheets for the end of the twenty-first century is still a topic of debate, especially due to model’s limitations in simulating land-ice processes^8,9^, the mid-Pliocene is considered a useful analogue for the end-of-century climate^10^.

Rainfall proxy archives do not exist for the tropics and Southern Hemisphere subtropics in the mid-Pliocene. However, paleo reconstructions of ice sheets, SSTs, vegetation distribution, soils and lakes performed by the Pliocene Research, Interpretation and Synoptic Mapping (PRISM) project^1^ are available to force climate models^4,12^ in order to estimate how rainfall may have looked in the mid-Pliocene. In this way, due to similarities between the mid-Pliocene and scenarios of future projected warming the Pliocene Model Intercomparison Project (PlioMIP) initiative was developed towards a better understanding of its climate scenario. To date, there has been no detailed investigation of Southern Hemisphere rainfall changes in the mid-Pliocene. Most studies have focused on the Northern Hemisphere monsoon systems^13–15^ or global land monsoons^16^. These studies reported intensified western African, Asian and Australian monsoons and a weakened South American monsoon.

SST changes indicated by both PlioMIP ensembles and proxy-data from the mid-Pliocene (Fig. 1a) suggest possible changes in the atmospheric circulation through changes in the meridional heat transport. These data clearly indicate a Northern Hemisphere warmer than Southern Hemisphere during the mid-Pliocene (Fig. 1a,b). Theoretical and observational studies indicate that in response to changes in the interhemispheric SST gradient, there is strengthening of the cooler hemisphere Hadley cell and displacement of the Inter-Tropical Convergence Zone (ITCZ) towards the warmer hemisphere^17,18^. In addition, changes in the intensity and position of the Hadley Cells and low-level winds can have a substantial impact on rainfall at subtropical latitudes. Indeed, Southern Hemisphere tropics and subtropics are the only regions that are simulated to be drier in the wetter atmosphere of the mid-Pliocene (Fig. 1c).

A closer examination in the tropical and subtropical Southern Hemisphere (Fig. 1d) indicates that reduced rainfall in the subtropical Southern Hemisphere is also related to a minimum SST increase. In the subtropical South Pacific, there is a strengthening of rainfall in the west and a weakening in the central-east coincident with SST changes. A warmer tropical Northern Hemisphere across all basins is also consistent with its increased rainfall due to the thermodynamical effect^19,20^ (‘wet-wetter’ theory). Furthermore, the non-uniform tropical warming results in increased tropical inter-hemispheric SST gradient, indicating that dynamical changes could also have contributed to changes in rainfall. As such, local changes in SST may also play a role in drying the subtropical Southern Hemisphere climate and amplifying the large-scale inter-hemispheric contrast.

The largest changes in rainfall in the Southern Hemisphere are likely associated with changes in the Inter-Tropical Convergence Zone (ITCZ) and Subtropical Convergences Zones (STCZs; Fig. 1d). In modern Southern Hemisphere climatology more than 50% of the annual rainfall occurs during the extended austral summer period (November to March) in most regions^21,22^. The ITCZ and SPCZs largely contribute to this. Firstly, the ITCZ that moves south during austral summer and increases rainfall in the Southern Hemisphere Tropical regions^21^. In addition, the STCZs are a summertime feature of the subtropics that can have rainfall rates comparable to the ITCZ^23^ (up to ~ 400 mm month^−1^).

The STCZs are bands of intense cloudiness and convection that set up transversally from tropical latitudes to the subtropics, commonly visible via satellite images. They develop when troughs of the subtropical jet penetrate the subtropics and poleward low-level winds prevail at the western limit of the subtropical highs^24–26^. In addition, these systems become intensified when moisture fluxes from the ITCZ and monsoon systems feed into the northern portion of the STCZs^27–29^. These convergence zones are more intense in the Pacific and Atlantic basins, namely the South Pacific Convergence Zone (SPCZ) and the South Atlantic Convergence Zone (SACZ), respectively, while they are less notable in the Indian Ocean^26,27^.

Here we examine model output from the Pliocene Model Intercomparison Project phases 1^12^ (PlioMIP1; 8 models; Table S1) and 2^4^ (PlioMIP2; 13 models; Table S1) and perform sensitivity experiments using the Community Atmospheric Model version 4 (CAM4; a component of one of the PlioMIP models) to understand the drivers of Southern Hemisphere summertime rainfall patterns during the mid-Pliocene.

## Results

**Figure 2:**
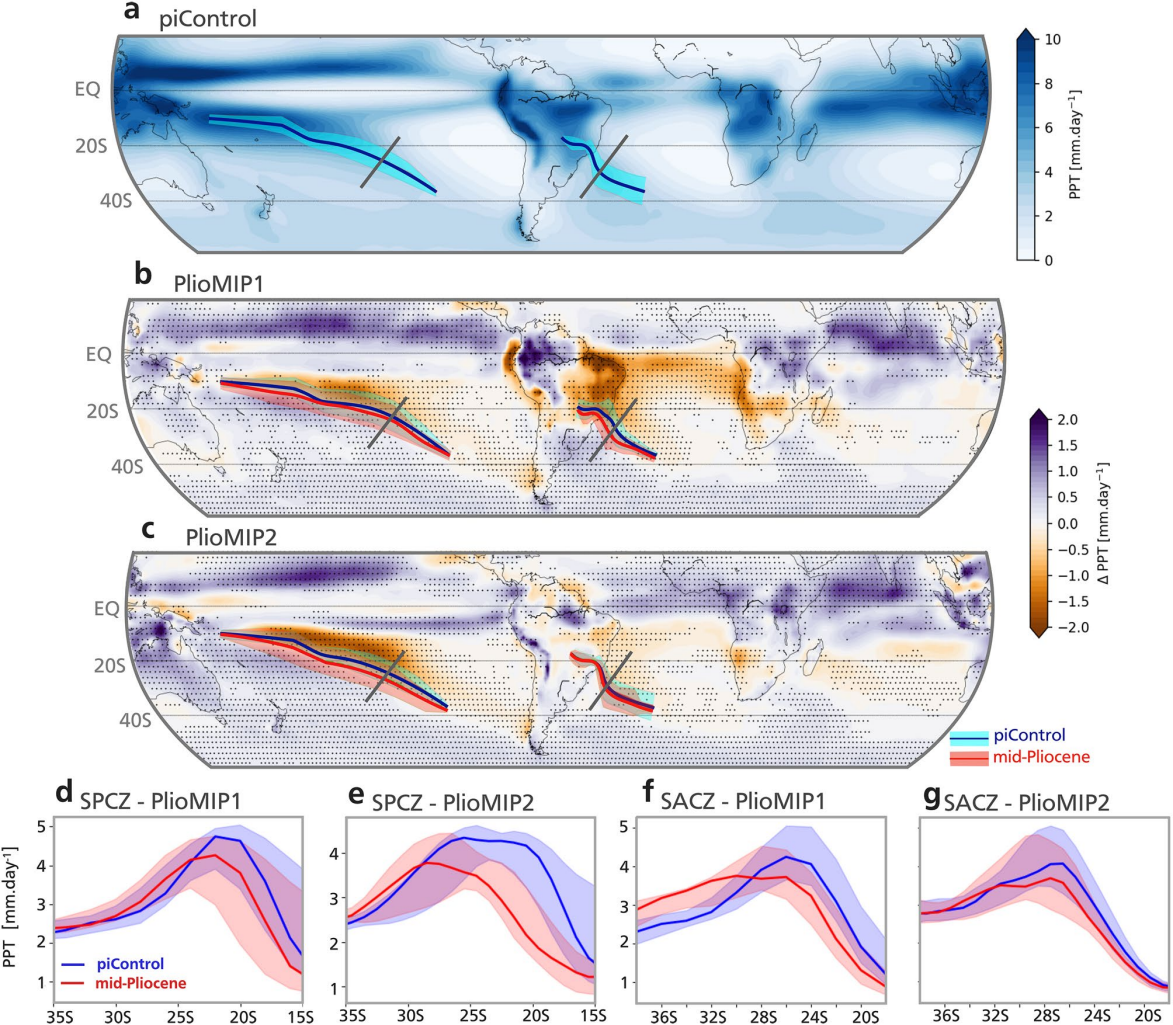
Summertime rainfall change and Subtropical Convergence Zones. (**a)** Multi-model mean November-to-March precipitation in the PlioMIP2 pre-industrial control simulation. Dark blue line indicates the position of the SPCZ and SACZ in the Pacific and Atlantic oceans, respectively. Banding indicates standard deviation range. Grey lines indicate the position of the cross section whose results are shown in the lower panels. (**b)** Multi-model mean change in the southern hemisphere precipitation from PlioMIP1 simulations relative to their pre-industrial control. Dark blue (red) lines indicate the position of the SPCZ and SACZ in the pre-industrial control (mid-Pliocene) simulation. (**c)** as ‘b’ but for PlioMIP2. Stippling indicates 72% model agreement in the sign of the change for PlioMIP1 and 75% for PlioMIP (see Methods). Panels (**d**) to (**g)** show the rainfall change along the cross sections indicates in panels ‘a’ to ‘c’. (**d)** and (**e)** show the change in SPCZ cross section in PlioMIP1 and 2, respectively. (**f)** and (**g)** as per ‘d’ and ‘e’ but for SACZ. Banding indicates interquartile range.

The PlioMIP simulations show that one of the most notable changes in the Southern Hemisphere summertime rainfall in the mid-Pliocene compared to pre-industrial conditions occurs in the subtropical regions along the STCZs (Fig. 2). Reduced precipitation is simulated along portions of both the SPCZ and SACZ (Fig. 2b,c) and a clear southward shift of these bands is seen (Fig. 2d–g; see Methods for how the position of the STCZs are obtained; for individual models see Supplementary Fig. S1, S2). Another change is associated with a northward shift of the ITCZ due to consistent increased rainfall in the Northern Hemisphere tropics, which is best observed in the Pacific Ocean for both PlioMIP phases (Fig. 2b,c).

The total November-to-March mean rainfall along the STCZs decreases in both PlioMIP1 and PlioMIP2 models (Supplementary Fig. S3). All PlioMIP1 and PlioMIP2 models exhibit a weakened SPCZ (− 6% to − 11% and − 10% to − 16%, respectively; numbers define the interquartile range; see Methods). The SACZ weakening is only evident in the PlioMIP1 (− 9% to − 15%) while in the PlioMIP2 the change is not as obvious (− 3% to 6%). It is important to note that the SACZ comprises land and ocean sectors. The land sector is more dependent on moisture fluxes from the Amazon and evapotranspiration processes, while the ocean sector is more dependent on the subtropical high system^30^. In the PlioMIP2 the changes in the SACZ ocean sector are more consistent than in the land sector (Fig. 2c). The reduced rainfall along the STCZs indicates weakened deep convection at these regions. The STCZ in the Indian Ocean, namely the South Indian Convergence Zone (SICZ), represents a less robust feature of the observed Southern Hemisphere monsoon system^27^ and, thus, a clear SICZ is usually not well seen in observations nor simulated in most climate models (Figs. 2a, S1,S2).

In addition to a weakening, the Southern Hemisphere STCZs significantly shifts southwards in both PlioMIP1 and PlioMIP2 models (Fig. 2d,e,f,g). In all models, the SPCZ displaces southwards (Fig. 2d,e): in the PlioMIP1 by − 1.9° to − 0.8°, and in the PlioMIP2 by − 4.1° to − 1.2°. The poleward shift in the SACZ position is more extreme in the PlioMIP1 models with a displacement of − 4.5° to − 0.6° compared to − 1.3° to − 0.4° in the PlioMIP2 simulations (Fig. 2f,g).

### Subtropical high intensification

Two important features that regulate the intensity, duration and position of the STCZs are the position of the subtropical jet and the strength and the location of the atmospheric subtropical high pressure systems^24–26,31^. Therefore, one may suppose that the weakening and southward shift of the STCZs in mid-Pliocene are related to a southward shift in the position of the subtropical jet. In this scenario, we would expect less penetration of the subtropical jet troughs at subtropical latitudes. This weakens the zonal pressure gradient that is necessary for the occurrence of poleward low-level flows^24^. However, we find that the subtropical jet shifts equatorward by a small amount in PlioMIP1 (0° to 0.6°; Table S3) while the PlioMIP2 models do not show uniform changes (− 0.1° to 1.2°; Table S3). In contrast, Atmospheric General Circulation Models (AGCM) experiments forced with reconstructed mid-Pliocene SST field from PRISM have simulated a southward shift in the position of the Southern Hemisphere subtropical jet^16^, which would contribute to weakened STCZs in the mid-Pliocene.

**Figure 3:**
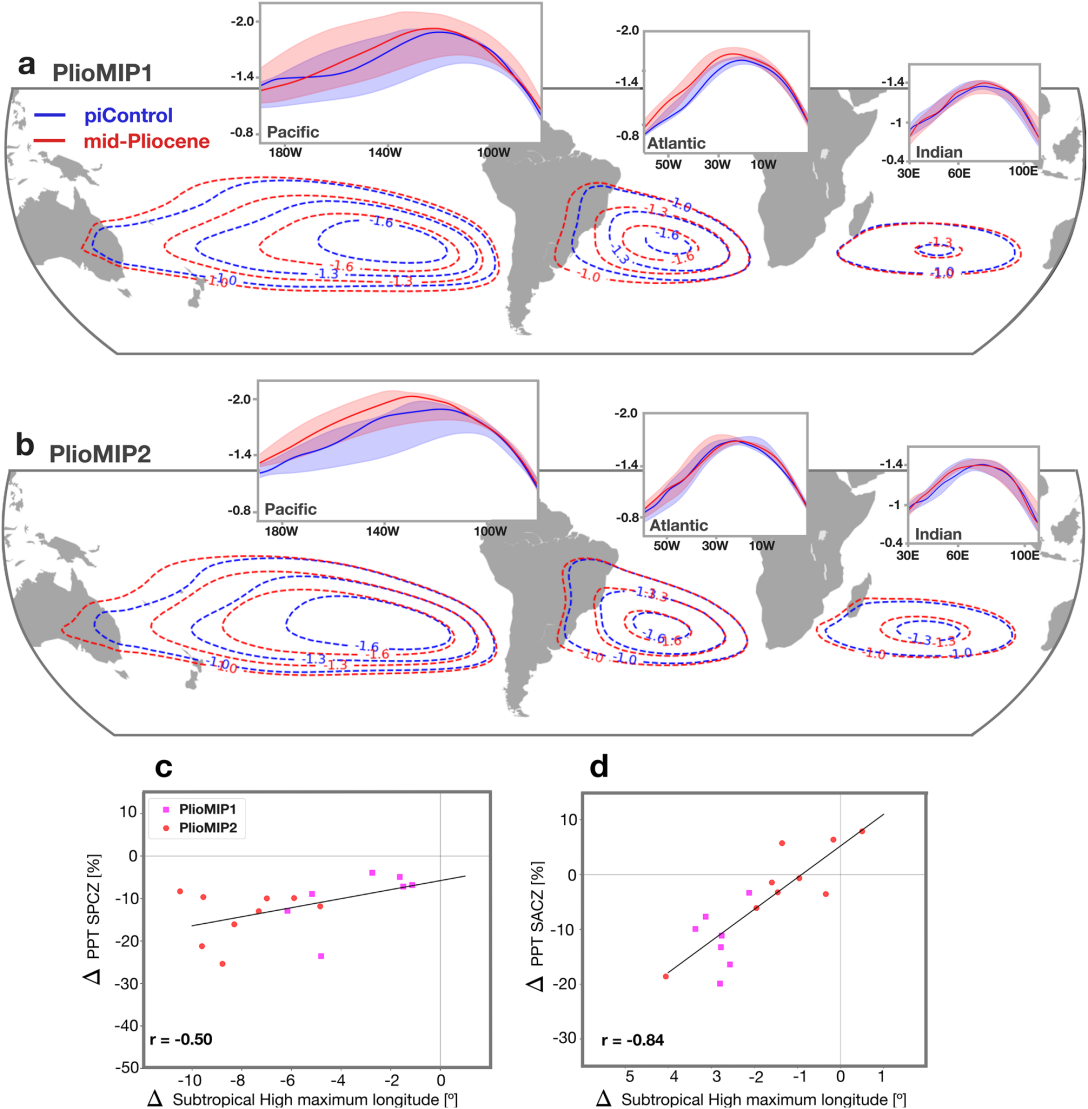
Subtropical high intensification. (**a)** PlioMIP1 MMM stream function at 850 hPa. Units 10^–7^ m^2^ s^−1^. Inner panels show the multi-model median stream function at the latitude of the maximum stream function for the Pacific, Atlantic and Indian oceans (Methods). Banding indicates inter-quartile range. (**b)** as ‘a’ but for PlioMIP2. (**c)** relationship between the change in the position of the subtropical high maximum and the change in the SPCZ precipitation along its diagonal sector. The subtropical high maximum position is computed as the weighted average longitude of the 850 hPa stream function shown in the inner panels of ‘a’ and ‘b’ (see methods). (**d)** as per ‘c’ but for SACZ.

The second cause for the STCZ changes might relate to an expansion and/or intensification of the Subtropical High pressure systems. An expanded Subtropical High prevents intrusions of the subtropical jet and weakens the continental heat lows that occur during summer months and contribute to an intensified zonal pressure gradient^31^. Quantifying the intensity of the subtropical highs is not a simple task when dealing with different climate backgrounds (+ 2–3 °C) as the global pressure weakens in a warmer atmosphere^19^. As such, we compute the stream function at 850 hPa to identify the position and intensity of the pressure systems at low levels relative to the background state (c.f. Methods). We find that the Subtropical High pressure systems, were more intense in the mid-Pliocene in both PlioMIP phases (Fig. 3a,b).

Across all models there is a strong relationship between the change in the position of the Subtropical High and the change in the intensity of the STCZs (Fig. 3c,d), where the models that simulate larger westward displacements of the subtropical high tend to be associated with larger rainfall reduction along both STCZs. The SPCZ comprises two sectors: a western zonal sector, which sometimes merges with the ITCZ in the western Pacific Warm Pool; and, an eastern NE to SW oriented sector^32^, extending south-eastward to Cook Islands. Here, we only found significant correlations associated with rainfall changes in the eastern sector. Changes in rainfall at the western SPCZ may be more influenced by changes in the position and temperature of the Western Pacific Warm Pool.

The South Pacific Subtropical High intensification and westward shift is more severe in the PlioMIP2 simulations (Fig. 3) with a westward shift of 7° to 9.5° compared to a shift of 1.5° to 5° in the PlioMIP1 simulations. In the Atlantic basin results for both PlioMIP phases show a westward shift; however, with larger values for the PlioMIP1 (− 2.7° to − 3°) compared to the PlioMIP2 (− 0.3° to − 1.6°).

One may question whether the STCZs changes are associated with changes in local SSTs^33^. In the South Atlantic basin, there is no clear agreement between the changes in precipitation and SSTs (Fig. 1d). In fact, the local SST in the southwestern South Atlantic has been shown to be a response to the SACZ^34^, and acts to dampening the SACZ strength^35^. In contrast, the South Pacific SST changes project onto the SPCZ location, suggesting that local forcing may have played a role in the Pacific convergence zone (Fig. 1d). However, it is difficult to evaluate whether the regional SST change is the main driver of the SPCZ changes as significant changes. The local SST could be a result of significant changes in winds more broadly, associated with the subtropical high system (Fig. 3), through the wind-evaporation-SST (WES) footprinting mechanism^36^.

### ITCZ-monsoon-STCZs relationship

Observational studies have shown that when moisture fluxes from the ITCZ and monsoons can penetrate into the STCZs, it can result in more intense deep convection at the convergence zone^21,27,29^. Although the SPCZ and SACZ are formed regardless of the influence of the ITCZ and monsoon systems^24,37^.

A coherent northward shift in the Pacific ITCZ is simulated in both PlioMIP phases. The austral summer position of the Pacific ITCZ moved northwards by + 0.9° to + 1.8° in the PlioMIP1 models and by + 1° to + 2.4° in the PlioMIP2 models (Table S3). All PlioMIP1 models show a northward ITCZ shift in the Atlantic of + 0.4° to + 2°, while in the PlioMIP2 this change is less clear (− 0.1° to + 1.3°).

**Figure 4:**
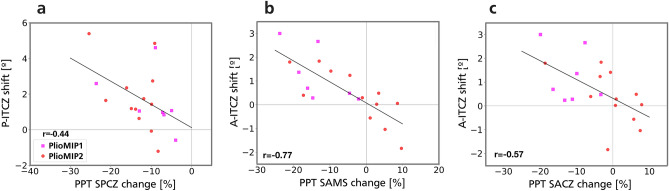
ITCZ-Monsoon-STCZs relationship. (**a)** relationship between the Pacific ITCZ shift and the change in the SPCZ precipitation along its diagonal section. (**b)** relationship between the Atlantic ITCZ shift and the mean change in the South American Monsoon system (SAMS) precipitation (25°S–10°S; 35°W–65°W). (**c)** relationship between the Atlantic ITCZ shift and change in the precipitation along the SACZ.

The change in the Pacific and Atlantic ITCZ positions are linearly related to the changes in rainfall along the diagonal sector of the SPCZ and the SACZ, respectively (Fig. 4). Here, we only found a significant relationship of the ITCZ with the most remote part of the SPCZ (diagonal sector; Fig. 4a) and not the section of the SPCZ that comes into more direct contact with the ITCZ (zonal sector). This is likely due to an indirect effect via atmospheric changes from equator to the subtropics and is different from the direct mechanism proposed earlier^32^. The ITCZ is a large-scale atmospheric feature whose position is tied to the meridional atmospheric circulation and, thus, meridional heat fluxes (discussed below). As such, the ITCZ migrates northwards as the southward energy flux at the equator (also called “energy flux equator”) intensifies. These changes intensify the Southern Hemisphere Hadley circulation and the Subtropical High system, which in its turn is closely linked to the diagonal sector of the SPCZ (Fig. 3c).

Furthermore, it is worth noting that in the Pacific region the ITCZ, Australian Monsoon and SPCZ are not all necessarily attached to one another. For example, two models (NorESM-L in PlioMIP1and CCSM4 in PlioMIP2) that show extreme northward shifts of the Pacific ITCZ (4.6° and 4.8°, respectively; outliers in Fig. 4a) do not weaken the SPCZ accordingly (− 9% and − 9%, respectively) but show great intensification of the Australian Monsoon (26% and 33%, respectively; all values in Table S3). This means that the Australian Monsoon can still intensify and provide moisture to the SPCZ even if the ITCZ shifts northward.

In the Atlantic sector, the moisture flux from the ITCZ to the SACZ is more direct than in the Pacific sector due to the topographical characteristics of South America^21,30^. During summertime, the ITCZ moisture flux flows into the Amazon basin up until reaching the Andes Mountain chain in the western Amazon. This moisture flux is then guided by the Andes towards south-eastern South America establishing the South American Monsoon System (SAMS)^30,38^. As such, a northward shift of the Atlantic ITCZ during summertime directly implies in weakened SAMS (r = 0.77; Fig. 4b; all values in Table S3). In this way, a more northward ITCZ position also suppress the equatorial moisture flux from reaching subtropical latitudes and, thus, weakens the SACZ system (r = 0.57; Fig. 4c).

The PRISM reconstruction provides further evidence of a northward ITCZ shift in the mid-Pliocene. It is well established that variations in tropical Atlantic SST directly influences the position of the ITCZ and precipitation in west African countries^39^ and Northeast Brazil^40^. The PRISM includes three high-level confidence sites in the north-western African upwelling region (Fig. 1a,d). These sites reveal an SST warming in between 1 °C to 2.2 °C relative to the Common Era (~ 1850). This indicates weakened upwelling in this region which in turn is suggested to be linked to decreased north-eastern trades winds due to a northward displacement of the Atlantic ITCZ^39^.

### Large-scale forcing

The similarity of changes across the ocean basins (Subtropical High intensification and northward ITCZ shift) suggests a common large-scale driver. Changes in the inter-hemispheric heat contrast have been shown to affect tropical and subtropical atmospheric circulation through changes in the meridional heat flux^17,18,39,41–43^. To evaluate whether the atmospheric changes in the PlioMIP models are consistent with these earlier studies, we performed sensitivity experiments using the National Centre for Atmospheric Research (NCAR) Community Atmospheric Model version 4 (CAM4). The CAM4 model is forced with multi-model mean monthly climatology of SST and sea-ice from PlioMIP1 and PlioMIP2. Land areas (i.e. land-ice, vegetation and soils) and CO_2_ concentrations were kept as pre-industrial (see Methods). We also forced CAM4 with model-based SST and sea-ice from PlioMIP pre-industrial controls as a comparison.

The AGCM experiments show qualitatively similar rainfall changes to those simulated by the fully-coupled PlioMIP models (Supplementary Fig. S4). The experiments were able to capture the large-scale changes in rainfall in the tropics and subtropics, particularly in the Pacific and Atlantic Oceans associated with the STCZs, and in most of South America and Southern Africa. Disagreements occur over regional areas in Western Africa, Tropical Indian Ocean and Australia. It is important to note that the PlioMIP models include prescribed changes in vegetation (PlioMIP1 and PlioMIP2) and soils (PlioMIP2 only; Table S2) and our AGCM experiments do not. Therefore, our experiments indicate that a large part of the mid-Pliocene precipitation change is associated with large-scale changes in SST and sea-ice (Supplementary Fig. S4).

**Figure 5:**
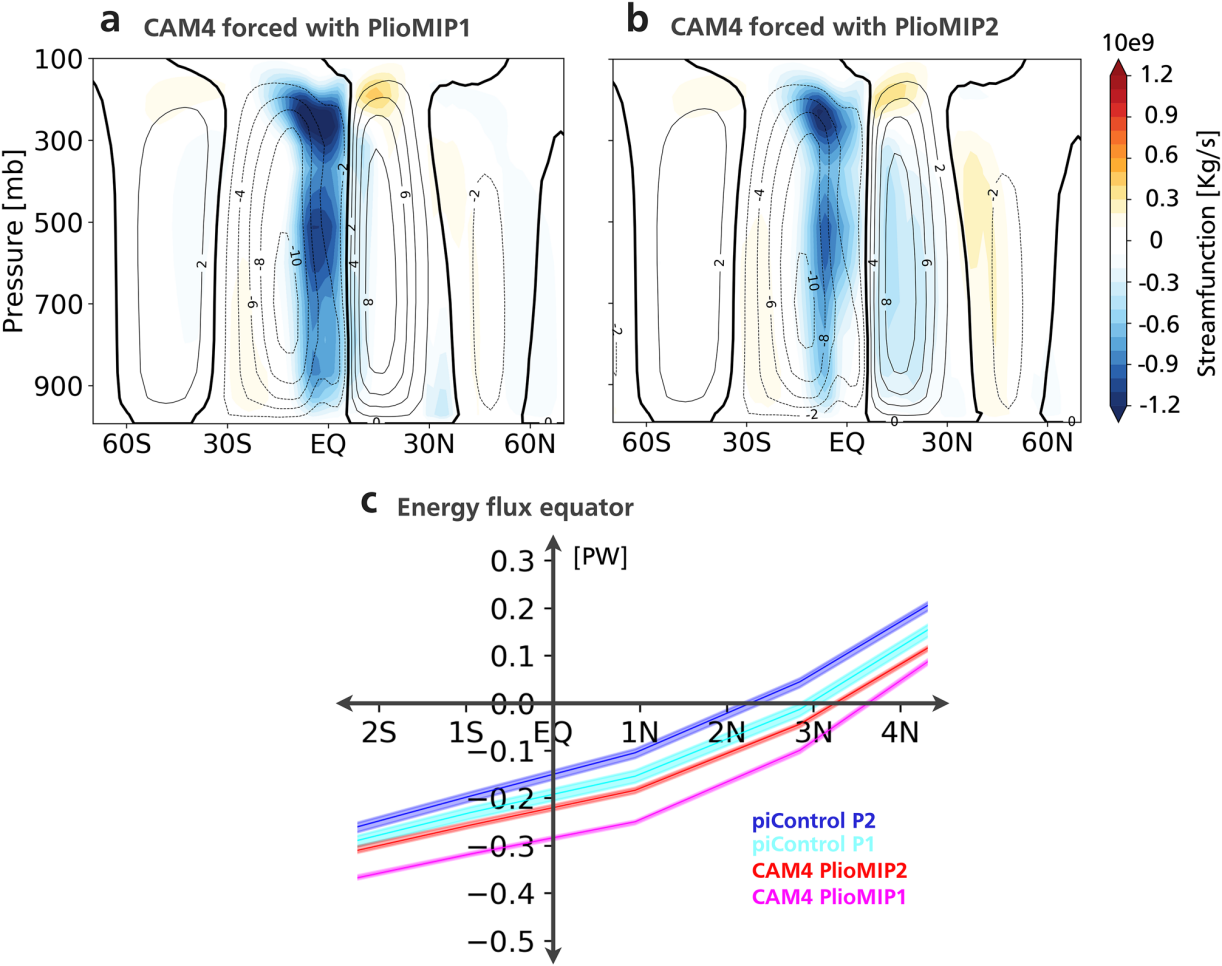
Atmospheric Meridional circulation changes. **(a)** 5-member ensemble mean of the zonally averaged meridional stream function simulated by the CAM4 model forced with model-based PlioMIP1 SST and sea-ice. Contours indicate piControl stream function: contour interval 2 × 10^–9^. Shading indicates change (mid-Pliocene minus piControl). Units: 10^–9^ kg s^−1^. (**b)** as ‘a’ but for the simulation forced with model-based PlioMIP2 SST and sea-ice. (**c)** integrated atmospheric meridional heat transport. Banding indicates ensemble range.

The changes in meridional circulation are in agreement with the PlioMIP simulations (Fig. 5). Changes in the zonal mean meridional stream function indicates an intensification and northward expansion of the Southern Hemisphere Hadley cell, while its Northern Hemisphere counter-part weakens (Fig. 5a,b). This results in a northward ITCZ displacement and increased cross-equatorial southward energy flux (CAM4-PlioMIP1: 0.09PW or 48%; CAM4-PlioMIP2: 0.07PW or 44%; Fig. 5c). The energy flux across the equator in our forced pre-industrial experiments agrees with the observed value of − 0.2 PW^44^ (− 0.2 PW forced with PlioMIP1 and − 0.16PW forced with PlioMIP2; Fig. 5c). These changes are consistent with a warmer Northern Hemisphere compared to Southern Hemisphere as indicated by proxy data and models (Fig. 1a,b) and enhanced Southern Hemisphere high pressure systems, which likely drive weakened STCZs.

## Discussion

A warmer atmosphere holds more moisture and as such would be expected to be associated with more rainfall. Indeed, all PlioMIP models increase global mean rainfall in the mid-Pliocene. However, the mid-Pliocene simulations show large spatial differences in rainfall and a mostly drier tropical and subtropical Southern Hemisphere.

**Figure 6:**
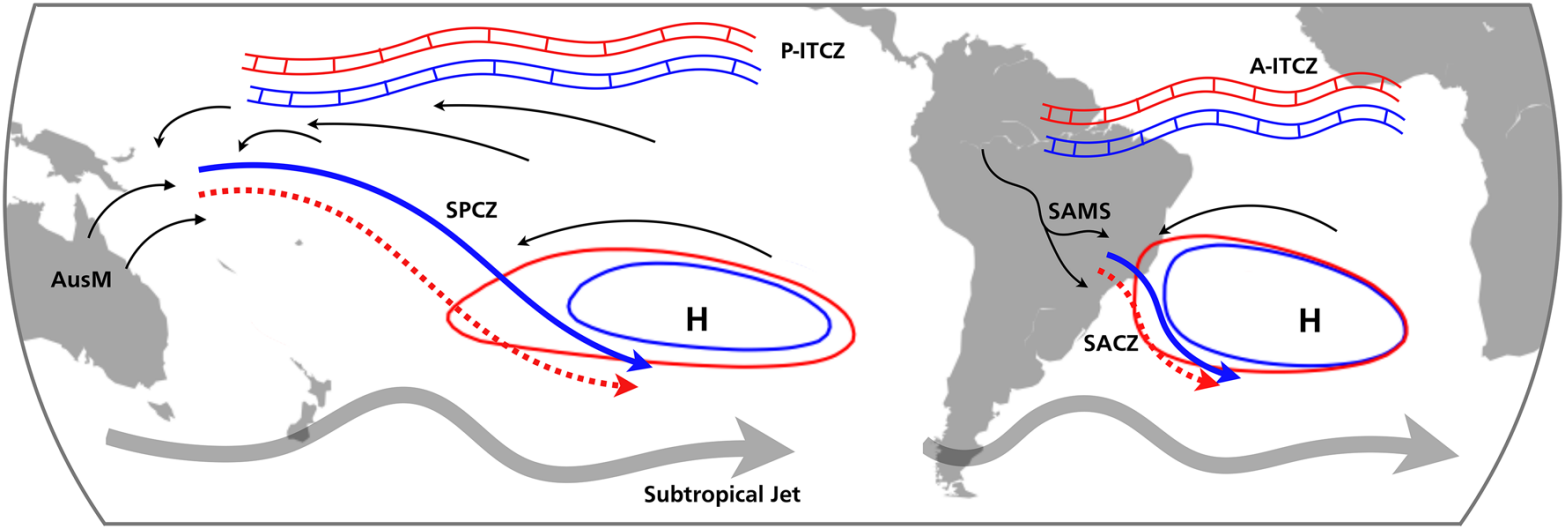
Schematic of the drivers of reduced rainfall in the Southern Hemisphere: subtropical high, ITCZ, STCZs and monsoon systems. Thicker grey arrows indicate the position of the subtropical jet. Thin black arrows indicate sources of moisture to the STCZs. AusM: Australian Monsoon. SAMS: South American Monsoon System. P-ITCZ: Pacific ITCZ. A-ITCZ: Atlantic ITCZ.

Our analyses provide a consistent picture of changes associated with the Southern Hemisphere summertime rainfall in the mid-Pliocene (Fig. 6). PlioMIP simulations indicate that the convergence zones in the South Pacific and South Atlantic weakened and displaced south during mid-Pliocene relative to pre-industrial conditions. These changes were associated with changes in interhemispheric SST gradient and consequently differences in large-scale atmospheric circulation. In particular, during the mid-Pliocene the northern hemisphere was warmer than the southern hemisphere. This interhemispheric temperature difference enhanced the southward energy flux equator as well as intensified the Southern Hemisphere Hadley circulation. As a result, the ITCZ shifted northward, and the Subtropical Highs in the Southern Hemisphere strengthened during the mid-Pliocene. Since the Subtropical Highs are related to the formation and location of the convergence zones, the intensified Pacific and Atlantic Subtropical Highs weakened the SPCZ and SACZ. As the ITCZ moves northward, the moisture inflow from the tropics to the subtropics over South America decreases, leading to a drier-than-normal SACZ. The ITCZ-SPCZ relationship is more complex and probably illustrates the link between the SPCZ weakening with changes in the large-scale circulation.

In this study, we suggest that the asymmetric warming between the two hemispheres may contribute to the tropical and subtropical changes in precipitation. In the PlioMIP experiments there is a large imposed change in the polar ice distribution. The change is considerably larger in the northern hemisphere compared to the southern hemisphere and gives rise to a large-scale asymmetric change in temperature^45^. Previous studies have shown that such an asymmetric change can give rise to an ITCZ shift ^17,18^ (and references therein). Additionally, local SST appears to play an important role in the ITCZ shift. However, the coupled changes to SST, atmospheric circulation and precipitation within the tropics could be in turn a response to large-scale remote changes. SST and precipitation are strongly coupled in the tropics and ocean–atmosphere feedbacks could have played an important role in both amplifying (i.e. WES and upwelling^39^) and damping (i.e. tropical ocean circulation^46^) ITCZ shifts. Thus, tropical changes in rainfall are also consistent with an increased tropical inter-hemispheric SST gradient^47^ (Fig. 1b,c,d). Another confounding factor that may have contributed to changes in local precipitation within the tropics is the expansion of the tropical rainforests in the PlioMIP simulations. For instance, a northward expansion of the tropical vegetation in Africa is known to increase precipitation in the Northern Hemisphere tropics, decrease trade winds and, thus warm the tropical North Atlantic^48^. It is important to note that the PlioMIP initiative focuses on a single interglacial time slice (KM5c at ~ 3.2 Ma) within the mid-Pliocene Warm Period^4^ (3–3.3 Ma). Thus, it is not possible to evaluate the sequencing of events that have triggered the mid-Pliocene rainfall shifts within the PlioMIP. Additionally, the relative effect of local versus remote SST forcings on tropical and subtropical rainfall during the mid-Pliocene requires further investigation.

The SACZ changes are less intense in the PlioMIP2 simulations than in PlioMIP1. A major difference between PlioMIP phases is that the second phase includes changes in soils, while the first phase uses modern-day distributions (PlioMIP phases 1 and 2 boundary conditions are described in Table S2). The PRISM reconstruction indicates a southward expansion in the tropical rainforests. As a consequence, this change is more evident in PlioMIP1 than in PlioMIP2^49^ (i.e. modern soils at subtropical latitudes used in PlioMIP1 are mostly replaced with soils from tropical rainforests which likely add a source of moisture in the subtropics in PlioMIP2). Both phases apply the same changes in vegetation. These regional differences in soils may offset the large-scale forcing, especially in South America where the SACZ is located mostly over the continent^30,50^. It is worth noting that the changes in vegetation (both phases) and soils (PlioMIP2 only) do not increase rainfall in south-eastern South America as one would expect due to the southward expansion of the Amazon rainforest. The effect of different soil types from PlioMIP1 to PlioMIP2 is clear over central Brazil: 100% of the PlioMIP1 models show weaker SAMS, while there is less agreement among PlioMIP2 with only 55% models showing reduced SAMS (Table S3). These regional changes could also be related to the spread among models with respect to the northward shift in the Atlantic ITCZ in the PlioMIP2.

Here, in addition to thermodynamically induced rainfall changes, changes in gradients drive shifts of important atmospheric features that result in a mostly drier subtropical Southern Hemisphere. This highlights that dynamical changes can be as important as thermodynamical ones in a warmer scenario. The mid-Pliocene (proxy and models) illustrates a scenario where the Northern Hemisphere warming rates are higher than its Southern counterpart. Over the past four decades, the Arctic has been warming faster than the global average^51^ with faster sea-ice melting than projected^52^. At the same time, the Antarctic warming has been increasing in the western sector while a weak cooling has been observed in the eastern sector^53^ similarly to the mid-Pliocene^5,6^. In this way the mid-Pliocene share some characteristics of the modern warming. The mid-Pliocene indicates that once differing warming rates between both hemispheres become important, dynamical atmospheric changes are likely to become relevant. In fact, the SACZ has been recently reported to be shifting poleward due to an intensified South Atlantic subtropical high over the satellite era (post-1979)^31^. Thus, the evaluation of the mid-Pliocene adds a constraint associated with differing rates of warming between hemispheres.

Limitations of the present study include the accuracy of proxy data used to force the models and climate model biases. Uncertainties remain in the extents of the Greenland ice sheet^4^ and the distribution of biomes (vegetation and soils), especially in South America due to a lack of geological sites^49^. Furthermore, climate models are thought to underestimate the magnitude of long-term warming due to a lack of polar feedback processes^54^. Indeed, most PlioMIP models underestimate the magnitude of the proxy-based polar amplification. As such, both proxy and modelling studies are needed for a better understanding of the mid-Pliocene climate. To isolate the influence of asymmetric interhemispheric temperature changes on the tropical circulation, future work could use partial coupling experiments where extratropical SST is prescribed, and the tropical ocean–atmosphere is allowed to evolve freely. For completeness, vegetation changes should also be taken into account.

## Methods

### Models and data

Models were selected according to data availability in the PlioMIP1 and PlioMIP2 databases. See supplementary Table S1 for a list of the models included in our analysis as well as variables used. A total of 8 PlioMIP1 and 13 PlioMIP2 models were analysed. PlioMIP1 and PlioMIP2 boundary conditions are specified in supplementary Table S2.

### Statistical significance of the changes

Statistical significance is based on model agreement in the sign of the change. This metric uses a binomial distribution of equal probability (p = q = 0.5) associated with an equal probability of a given model simulating a positive or negative change. For N = 7 (models) the significance level of 95% is achieved when at least 5 models (72%) agree on the sign of the change (Supplementary Fig. S5). For N = 9 the same significance is achieved with 7 models (77%) and for N = 12 this is 9 models (75%; Supplementary Fig. S5). All values cited correspond to the inter-quartile range (IQR). When indicating changes, if the IQR does not cross the zero value, it indicates that there is at least a 75% model agreement in the sign of the change. All values computed in this study are presented in supplementary Table S3.

### Position of the subtropical convergence zones

Calculated as the maximum rainfall latitude, at each longitude, between 15°S–40°S and 160°W–190°W (0–20°S and 160°E–160°W) for the SPCZ diagonal (zonal) sector and 55°W–20°W for the SACZ. The COSMOS model was not able to simulate the poleward diagonal projection of the STCZs in its pre-industrial simulations in either phases of the PlioMIP (Supplementary Fig. S1, S2) and, thus, we have not included it in our analysis.

### STCZs cross section positions

SPCZ: from 145°W/35°S to 165°W/15°S. SACZ: from 50°W/38°S to 30°W/18°S.

### Position of the subtropical jet

Weighted mean latitude of U-wind (at 200 hPa) based on latitudes where the intensity was greater than 50% of its maximum in the zonal mean.

### Intensity of the STCZs

Total November-to-March mean rainfall along the STCZ length.

### Position of the centre of the atmospheric subtropical highs

We first computed a zonal cross section in the latitude of the maximum stream function (at 850 hPa) value in the subtropics (20°S–40°S) which are shown in the inner panels of Fig. 3. In this cross section we, then, compute the weighted mean longitude based on longitudes where the intensity was greater than 90% of its maximum.

### ITCZ position

Precipitation weighted mean latitude based on latitudes where precipitation is greater than 50% of its maximum in the zonal mean. Pacific and Atlantic ITCZs are computed over their respective oceans only (land areas not included). This method may include double-ITCZ and double-ITCZ biases if the double-ITCZ associated precipitation is greater than 50% of the maximum.

### South American Monsoon strength

Total November-to-March precipitation in between 10–25°S and 35–65°W.

### Australian Monsoon strength

Total November-to-March precipitation in between 15–25°S and 130–150°E.

### CAM4 experimental design

The 2° resolution CAM4 version is used. A total of 4 experiments were performed each with a 5-member ensemble. Each member was run for 31 years. The first year of each member was excluded as it includes the atmospheric spin-up. Each member was initialized in a different starting date in order to have a different initial condition. Experiment 1: CAM4 forced with pre-industrial SST and sea-ice climatology based on multi-model mean PlioMIP1 control simulation. CO_2_ was set at its pre-industrial value of 280.4 ppmv. Experiment 2: CAM4 forced with mid-Pliocene SST and sea-ice climatology based on multi-model mean PlioMIP1 simulations. We have not included any changes over continental areas and the CO_2_ concentration was kept at its pre-industrial value. Experiments 3 and 4 consist of reproducing experiments 1 and 2, respectively, but with multi-model mean SST and sea-ice from PlioMIP2.

## Supplementary information

Supplementary information
