# Supplementary material for: Drier tropical and subtropical Southern Hemisphere in the mid-Pliocene Warm Period
Source: Sci Rep. 2020 Aug 10;10:13458. doi: 10.1038/s41598-020-68884-5 (PMC7417591; doi:10.1038/s41598-020-68884-5)
Supplement: Supplementary file 1 — Supplementary information [file 41598_2020_68884_MOESM1_ESM.docx]

Supporting Information for

**Drier Tropical and Subtropical Southern Hemisphere in the Mid-Pliocene Warm Period**

Gabriel M. Pontes^*1,2^, Ilana Wainer^1^, Andréa S. Taschetto^2,3^, Alex Sen Gupta^2,3^, Ayako Abe-Ouchi^4^, Esther C. Brady^5^, Wing-Le Chan^4^, Deepak Chandan^6^, Camille Contoux^7^, Ran Feng^5^, Stephen J. Hunter^8^, Yoichi Kamae^9^, Gerrit Lohmann^10^, Bette L. Otto-Bliesner^5^, W. Richard Peltier^6^, Christian Stepanek^10^, Julia Tindall^8^, Ning Tan^7,11^, Qiong Zhang^12^, Zhongshi Zhang^11,12,13^

^1^Oceanographic Institute, University of São Paulo, São Paulo, Brazil

^2^Climate Change Research Centre, The University of New South Wales, Sydney, Australia

^3^ARC Centre of Excellence for Climate Extremes, The University of New South Wales, Australia

^4^Centre for Earth System Dynamics, Atmosphere and Ocean Research Institute, University of Tokyo, Japan

^5^National Center for Atmospheric Research, Boulder, USA

^6^Department of Physics, University of Toronto, Toronto, Canada

^7^Laboratoire des Sciences du Climat et de l’Environnement, Université Paris-Saclay, France

^8^School of Earth and Environment, University of Leeds, Leeds, UK

^9^Faculty of Life and Environmental Sciences, University of Tsukuba, Tsukuba, Japan

^10^Alfred Wegener Institute, Helmholtz Centre for Polar and Marine Research, Bremerhaven, Germany

^11^Key Laboratory of Cenozoic Geology and Environment, Institute of Geology and Geophysics, Chinese Academy of Sciences, Beijing, China

^12^Department of Physical Geography and Bolin Centre for Climate Research, Stockholm University, Stockholm, Sweden

^13^NORCE Norwegian Research Centre, Bjerknes Centre for Climate Research, Bergen, Norway

*Corresponding author: Gabriel Pontes ([gabrielpontes@usp.br](mailto:gabrielpontes@usp.br))

**Contents of this file**

Figures S1 to S5

Tables S1 to S3

**Introduction**

This document provides additional information for the following analysis: (1) Position of the Subtropical Convergence Zones (STCZ) in each PlioMIP1 (Figure S1) and PlioMIP2 model (Figure S2); (2) Rainfall climatology of the STCZs (Figure S3); (3) comparison of the PlioMIPs multi-model means rainfall changes and the rainfall changes simulated with the CAM4 model (Figure S4); (4) additional information on the statistical analysis (Figure S5). In addition, table S1 lists the PlioMIP models used in this study as well as theirs respective variables. Table S2 specifies the boundary conditions used by PlioMIP1 and PlioMIP2 models. Finally, all results computed in this study are presented in Table S3.

**Figure S1 –** Position of the STCZs as simulated by each individual model participant of the PlioMIP1. Red: position in the pre-industrial control simulation; Green: position in the mid-Pliocene simulation.

**Figure S2 –** As per “Figure S1” but for PlioMIP2.

**Figure S3 – STCZs rainfall climatology. a)** MMM rainfall climatology along the SPCZ length in the PlioMIP2 pre-industrial control simulation. **b)** MMM change (mid-pliocene minus piControl) in the PlioMIP1 SPCZ climatology. **c)** as per ‘b’ but for PlioMIP2. ‘d’, ‘e’, and ‘f’ as per ‘a’, ‘b’ and ‘c’, respectively, but for SACZ. Stippling indicates 70% model agreement in the sign of the change.

**Figure S4 –** comparative of mean November-to-March rainfall changes simulated by the CAM4 model forced with PlioMIP multi-model mean SST and sea-ice with the rainfall changes simulated by the fully coupled PlioMIP models. a) 5-member ensemble mean rainfall change of the CAM4 model forced with PlioMIP1 multi-model mean and sea-ice. b) multi-model mean PlioMIP November-to-March rainfall change. c) as per ‘a’ but CAM4 forced with multi-model mean PlioMIP2 SST and sea-ice. d) as per ‘b’ but for PlioMIP2.

**Figure S5 –** Cumulative probability functions for binomial distributions with p = q = 0.5 and N = 7,9, and 12. In this study, ‘N’ is associated to the number of models used in each analysis.

**Table S1.** Models and variables used in the present study.

|  | **Precipitation** | **Winds** | **SST** | **Sea-ice concentration** |
| --- | --- | --- | --- | --- |
| **PlioMIP1** |  |  |  |  |
| CCSM4 | y | y | y | y |
| COSMOS | y | n | y | y |
| GISS-E2-R | y | y | y | y |
| HadCM3 | y | y | y | y |
| IPSL-CM5A | y | y | y | y |
| MIROC4m | y | y | y | y |
| MRI-CGCM2.3 | y | y | y | y |
| NorESM-L | y | y | y | y |
| **PlioMIP2** |  |  |  |  |
| CCSM4 | y | n | y | y |
| CESM1.2 | y | n | n | n |
| CESM2.1 | y | n | n | n |
| COSMOS | y | n | y | y |
| EC-EARTH3.1 | y | y | y | y |
| HadCM3 | y | y | y | y |
| IPSL-CM5A | y | y | y | y |
| IPSL-CM5A2 | y | y | y | y |
| MIROC4m | y | y | y | y |
| MRI-CGCM2.3 | y | y | y | y |
| NorESM-L | y | y | y | y |
| NorESM1-F | y | y | y | y |
| UofT-CCSM4 | y | y | y | y |

**Table S2.** PlioMIP1 and PlioMIP2 boundary conditions.

|  | LSM | Topo. | soils | lakes | Ice  Greenl. | Ice  Antarct. | vegetation | CO_2_ | Orbital  Parameters |
| --- | --- | --- | --- | --- | --- | --- | --- | --- | --- |
| Pre-industrial | Modern | Modern | Modern | Modern | Modern | Modern | Modern | 280 | Modern |
| PlioMIP1 (experiment 2) | Plio | Plio | Modern | Modern | Plio (~50% retreat) | Plio | Plio | 405 | Modern |
| PlioMIP2 (Eoi400) | Plio | Plio | Plio | Plio | Plio (~70% retreat) | Plio | Plio | 400 | Modern |

**Table S3 –** All values computed in this study. Sign of the change indicated by colours: blue (negative) and red (positive).

|  | | **PioMIP1** | | | | | | | | | | **PlioMIP2** | | | | | | | | | | | | | |
| --- | --- | --- | --- | --- | --- | --- | --- | --- | --- | --- | --- | --- | --- | --- | --- | --- | --- | --- | --- | --- | --- | --- | --- | --- | --- |
|  | | CCSM4 | | GISS-E2-R | HadCM3 | IPSLCM5A | MIROC4m | MRI-CGCM2.3 | NorESM-L | **75% model agreem.** | **100% model agreem.** | CCSM4 | CESM1.2 | CESM2.1 | EC-EARTH3.1 | HadCM3 | IPSL-CM5A | IPSLCM5A2 | MIROC4m | MRI-CGCM2.3 | NorESM-L | NorESM1-F | UofT-CCSM4 | **75% model agreem.** | **100% model agreem.** |
| Δ PPT [%] | SACZ | 16 | 13 | | 8 | 11 | 20 | 3 | 10 | **Y** | **Y** | 7 | 1 | 1 | 1 | 1 | 6 | 3 | 4 | 19 | 6 | 8 | 6 | **N** | **N** |
|  | SPCZ | 5 | 7 | | 24 | 13 | 4 | 7 | 9 | **Y** | **Y** | 9 | 15 | 14 | 21 | 25 | 13 | 10 | 8 | 10 | 16 | 10 | 12 | **Y** | **Y** |
| Δ Position [ºN] | SACZ | 1.6 | 0.1 | | 5.1 | 0.3 | 4.4 | 1.3 | 4.7 | **Y** | **N** | 1 | 0.9 | 0.8 | 0.1 | 1.2 | 2.1 | 0.4 | 0.5 | 5 | 0.4 | 1.6 | 0.1 | **Y** | **Y** |
|  | SPCZ | 1.9 | 1.5 | | 1.9 | 1.1 | 0.2 | 0.5 | 2 | **Y** | **Y** | 4 | 4.5 | 5.1 | 2.4 | 3.5 | 1.2 | 1.2 | 3 | 0.9 | 4.6 | 1 | 3.4 | **Y** | **Y** |
| Δ Subtropical High [ºE] | Atl | 2.6 | 2.8 | | 3.1 | 2.8 | 2.8 | 2.1 | 3.4 | **Y** | **Y** | - | - | - | 1.6 | 1 | 2 | 1.5 | 0.3 | 4.1 | 1.4 | 0.5 | 0.2 | **Y** | **N** |
|  | Pcf | 1.6 | 1.1 | | 4.8 | 6.2 | 2.7 | 1.5 | 5.2 | **Y** | **Y** | - | - | - | 9.6 | 8.8 | 7.3 | 7 | 10.5 | 9.5 | 8.3 | 5.9 | 4.8 | **Y** | **Y** |
| Δ ITCZ [ºN] | Atl | 0.7 | 0.2 | | 2.7 | 0.3 | 3 | 0.5 | 1.4 | **Y** | **Y** | 1 | 0.3 | 0 | 1.8 | 1.4 | 0.4 | 1.8 | 1.2 | 1.8 | 0.6 | 0.1 | 0.5 | **Y** | **N** |
|  | Pcf | 1.1 | 0.8 | | 2.6 | 1.1 | 0.6 | 1.1 | 4.6 | **Y** | **N** | 4.9 | 1.2 | 1.2 | 1.6 | 5.4 | 0.6 | 0.1 | 1.2 | 2.7 | 1.4 | 2.4 | 1.7 | **Y** | **N** |
| Δ Sub. Jet [ºN] |  | 0.2 | 0.5 | | 1.6 | 0.1 | 1 | 0.1 | 0.5 | **Y** | **N** | - | - | - | 0.4 | 0.1 | 0.2 | 0 | 1.8 | 1.8 | 0.4 | 0.1 | 0.1 | **N** | **N** |
| Δ AusM [%] |  | 12 | 6 | | 12 | 16 | 5 | 10 | 26 | **Y** | **N** | 33 | 18 | 7 | 45 | 51 | 7 | 8 | 11 | 8 | 25 | 26 | 14 | **Y** | **N** |
| Δ SAMS [%] |  | 16 | 2 | | 13 | 15 | 24 | 5 | 18 | **Y** | **Y** | 5 | 1 | 3 | 10 | 10 | 17 | 13 | 4 | 21 | 1 | 9 | 3 | **N** | **N** |
